# Bayesian sample size determination using robust commensurate priors with interpretable discrepancy weights

**DOI:** 10.1177/09622802261432816

**Published:** 2026-04-16

**Authors:** Lou E Whitehead, James MS Wason, Oliver Sailer, Haiyan Zheng

**Affiliations:** 1 Biostatistics Research Group, Population Health Sciences Institute, 5994 Newcastle University, UK; 2 84647 Boehringer Ingelheim Pharma GmbH & Co. KG, Germany; 3 Department of Mathematical Sciences, 1555 University of Bath, UK

**Keywords:** Bayesian sample size determination, commensurate priors, Historical borrowing, prior aggregation, uniform shrinkage

## Abstract

Randomized controlled clinical trials provide the gold standard for evidence generation in relation to the efficacy of a new treatment in clinical research. Relevant information from previous studies may be desirable to incorporate in the design and analysis of a new trial, with the Bayesian paradigm providing a coherent framework to formally incorporate prior knowledge. Many established methods involve the use of a discounting factor, sometimes related to a measure of ‘similarity’ between historical and the new trials. However, it is often the case that the sample size is highly nonlinear in those discounting factors. This hinders communication with subject-matter experts to elicit sensible values for borrowing strength at the trial design stage. Focussing on a method that can incorporate historical data from multiple sources, we highlight a particular issue of nonmonotonicity and explain why this causes issues with interpretability of discounting factors (hereafter referred to as ‘weights’). We propose a solution from which an analytical sample size formula is derived. We then propose a linearization technique such that the sample size changes uniformly over the weights. This leads to interpretable weights (as a percentage of information to borrow/discount) which could facilitate easier elicitation of expert opinion on their values.

## 1. Introduction

In clinical drug development randomized controlled trials (RCTs) are regarded as the gold standard for evaluating the efficacy of new treatments or interventions. Randomization of trial participants to the new treatment or a control group aims to reduce bias and provide a rigourous tool to examine whether a causal relationship exists between an intervention and outcome.^1^ Sample size calculations are an essential part of clinical trial design, with a sample needing to be at least large enough to meet the study objectives but also small enough to minimize (for example) ethical or cost concerns.^2^ In the frequentist paradigm, the number of participants recruited onto a study is often chosen to control the type I error rate (the rate of incorrectly declaring a treatment efficacious) and power (the rate of correctly declaring a treatment efficacious) to pre-specified levels, based on assumptions about the sampling distribution of the data and the size of the treatment effect considered clinically meaningful.

Designing a trial with a large enough sample size to achieve the frequentist power can sometimes be infeasible, especially when there are limited numbers of participants available. This might be the case, for example, in rare disease trials or trials in pediatric populations. Pre-trial information, from historical studies conducted under similar circumstances, or elicited directly from expert opinion, could be useful to overcome this challenge, with the Bayesian paradigm offering a powerful tool to formalize this approach. In the Bayesian framework, a prior distribution is formed for a parameter of interest, which is then updated by the observed data to form a posterior distribution from which inferences can be made. Instead of designing a trial around frequentist type I error rates and power, Bayesian designs rely on alternative metrics for success; for instance, specification of posterior decision thresholds (the level of confidence we desire to have that a treatment is efficacious or futile), or the width or coverage probabilities of Bayesian credible intervals. The application of Bayesian methodology for trial design to the specific areas noted above has been considered in the literature, for example, by Hampson et al.^3^ for trials in very rare diseases, and Wadsworth et al.^4^ for pediatric studies.

Neuenschwander et al.^5^ classify Bayesian methods for clinical trial design incorporating historical data according to the approach of constructing a prior distribution for a parameter of interest as follows:

- ‘Irrelevance’, where a prior is formed without reference to previous studies.
- ‘Similar’, also termed ‘exchangeable’, where a prior is formed by assuming that the parameter of interest in the new trial has been generated from the same underlying distribution as the parameter(s) in the historical trial(s). The meta-analytic predictive (MAP) prior proposed in Neuenschwander et al.^5^ is based on this assumption, with the authors noting the importance of careful selection of relevant historical data to render the exchangeability assumption plausible. A robust extension^6^ aims to effectively discount historical data in the case of prior/data conflict by using a weighted mixture distribution consisting of the MAP prior and a weakly informative component.
- ‘Equal but discounted’, which assumes parameters are the same, but discounts the precision of the parameter in the historical trial(s). The ‘power prior’ suggested by Ibrahim and Chen^7^ takes this approach, whereby historical evidence is downweighted by taking its likelihood to a power, aϵ[0,1] .
- ‘Biased’, which assumes historical parameters are potentially biased versions of the parameter in the new trial. The ‘commensurate prior’^8,9^ comes under this category, where historical information is downweighted by a commensurability parameter to form a predictive prior for the new study. The commensurability parameter directly parameterizes the similarity between each historical source and new data.
- ‘Equal’, equivalent to pooling historical data with the new study data.

The importance of carefully selecting historical trials to be included for planning a new trial is well understood. If the assumption of similarity is not satisfied, this can result in increased mean square error (MSE) of point estimates due to bias and either reduced power or increased type I error rate depending on the direction of the bias.^10^ Conversely, incorporation of quality historical information allows for reduced MSE and increased power (or reduced type I error rate) within the new trial. A seminal paper by Pocock^11^ provided a set of criteria for assessing the comparability between historical and current trials. Expert elicitation can play an important role in assessing comparability and helping to choose model parameters but the elicitation process is not trivial.^12^ Johnson et al.^13^ review different methods to elicit beliefs for Bayesian priors.

This paper focusses on the design of a new two-arm RCT incorporating historical data from similar RCTs. We follow the series of research in sample size determination based on ‘commensurate priors’ in Zheng et al.^14^ in which the use of discrepancy weights ϵ[0,1] quantifying the probability of (ir)relevance of information from multiple historical sources (with respect to the new trial) was recommended. The methodology in Zheng et al.^14^ was later extended to basket trials in Zheng et al.^15^. In the setting of borrowing from historical data, specification of study-specific discrepancy weights at the design stage provides an explicit opportunity to make judgments concerning the relevance and rigour of past studies with respect to the new study.^5^ Furthermore, the elicitation of study-specific discrepancy weights may be more intuitive than eliciting model parameters of a distribution.

It would be desirable that the discrepancy weights recommended in Zheng et al.^14^ act uniformly with respect to the amount of information that would subsequently be incorporated from a particular source. For example, specifying a historical study-specific weight of 0.50 should result in incorporation of 50% of the information from that source into the new trial design. In Section 2 we demonstrate that this is not the case, and the weights in fact exhibit undesirable highly nonlinear behaviour. Of primary concern is nonmonotonicity, caused by the method used to aggregate information from multiple sources into a single prior, which hinders interpretability and makes elicitation of such weights difficult. Additional nonlinearity is also an issue, whereby small values of weights result in faster changes in the amount of information incorporated into the prior than their complement. We propose a solution in two parts. Firstly, in Section 3, an alternative method of prior aggregation is proposed, for which the nonlinearity then has a simpler pattern, and from which a Bayesian sample size formula is derived. Secondly, a technique for linearization is provided such that the weights provide uniform shrinkage with respect to the sample size. The aim is to make interpretability simpler and thereby facilitate easier elicitation of such values. Section 4 provides a motivating example in which a sample size is sought for a hypothetical new RCT using historical data from several real-life historical clinical trials. Section 5 presents a brief simulation study confirming pre-specified statistical properties are preserved across a range of scenarios with sample sizes determined according to our method. We finish with a discussion highlighting areas for future research in Section 6.

## 2. Problem formulation

Consider planning a two-arm randomized controlled superiority trial (referred to as ‘new trial’ in the following) to evaluate an investigational treatment or intervention. Let $Y_{ij}$ be the measured post-randomization outcomes in the new trial for patient $i=1,\ldots ,n_{j}$ in treatment group j=T,C . Explicitly, j=T refers to the experimental treatment group and j=C refers to the control group. We assume outcomes are normally distributed with common variance in the outcome measures such that $Y_{ij}∼N(\mu_{j},\sigma_{0}^{2})$ . The groupwise sample means therefore follow a normal distribution, ${\bar{Y}}_{j}∼N(\mu_{j},(\sigma_{0}^{2}/n_{j}))$ . Considering the distribution of the difference in group means leads to

$${\bar{Y}}_{T}-{\bar{Y}}_{C}={\bar{Y}}_{\Delta }∼N\left(\mu_{\Delta },\frac{\sigma_{0}^{2}}{nR(1-R)}\right),$$

where the parameter $\mu_{\Delta }=\mu_{T}-\mu_{C}$ is the primary inferential target. $n=\sum_{j=T,C}n_{j}$ are the total number of trial participants randomized (to treatment or control) at the initiation of the trial and $R=n_{T}/n$ is the proportion randomly assigned to the experimental treatment arm.

In the Bayesian framework with no borrowing from historical data (for assumed known $\sigma_{0}^{2}$ ), a prior for $\mu_{\Delta }$ is specified,

$$\mu_{\Delta }∼N\left(\mu_{0},s_{0}^{2}\right),$$

where $\mu_{0}$ and $s_{0}$ are user-defined hyper-parameters (which might be chosen for example in the case of no prior information such that the prior is only weakly informative relative to the likelihood). The prior is then updated by the trial data to give a posterior distribution,

$$\mu_{\Delta }|y_{new}∼N\left(d_{\theta_{0}},\sigma_{\theta_{0}}^{2}\right).$$

The posterior mean is given by

(1) $$d_{\theta_{0}}=\frac{\mu_{0}\cdot s_{0}^{-2}+({\bar{y}}_{T}-{\bar{y}}_{C})\cdot nR(1-R)/\sigma_{0}^{2}}{s_{0}^{-2}+nR(1-R)/\sigma_{0}^{2}},$$

and the posterior variance is

(2) $$\sigma_{\theta_{0}}^{2}={\left(\frac{1}{s_{0}^{2}}+\frac{nR(1-R)}{\sigma_{0}^{2}}\right)}^{-1}.$$

### 2.1. Formulating priors from multiple historical sources

Suppose instead that there are Q sources of historical data, $y_{1},\ldots ,y_{Q}$ , that are relevant to incorporate in the planning of the new trial. $\lambda_{q}$ are the parameter counterparts of $\mu_{\Delta }$ in the historical trials and it is assumed they have been summarized by posterior distributions, $\lambda_{q}∼N(\theta_{q},\tau_{q}^{2})$ . Defining $\mu_{\Delta (q)}$ as the prediction for $\mu_{\Delta }$ in the new trial based on the information from trial q alone, a set of Q commensurate predictive prior distributions for $\mu_{\Delta }$ are formed centred on each $\theta_{q}$ ,

(3) $$\mu_{\Delta (1)}∼N(\theta_{1},\xi_{1}^{2}),\;\;\ldots ,\;\;\mu_{\Delta (Q)}∼N(\theta_{Q},\xi_{Q}^{2}).$$

We let $\xi_{q}^{2}=\tau_{q}^{2}+\nu_{q}^{-1}$ , where $\nu_{q}$ parameterizes the ‘commensurability’^15^ between $\lambda_{q}$ and $\mu_{\Delta }$ in terms of precision (further details are given in the following section).

### 2.2. Estimating $\xi_{q}^{2}$

To quantify the relevance of each historical data source in respect of the new experiment, Zheng et al. introduce discrepancy parameters, $w_{q}=\{w_{1},\ldots ,w_{Q}\}$ . The ‘discrepancy’ of interest, for a continuous parameter like treatment effect, is the mismatch in either the location or scale parameters, or both. That is, $w_{q}$ are prior weights ∈[0,1] intended to represent preliminary skepticism about how similar $\lambda_{q}$ (and/or $\tau_{q}^{2}$ ) and $\mu_{\Delta }$ (and/or $s_{0}^{2}$ ) are. Weights are incorporated into a Gamma mixture prior for the precision parameter, $\nu_{q}$ :

(4) $$\nu_{q}∼w_{q}\;Ga(a_{01},b_{01})+(1-w_{q})\;Ga(a_{02},b_{02}),$$

with $w_{q}\;\epsilon \;[0,1],\;\;a_{01},a_{02}>1$ . This mixture prior is favoured for robust inferences as it offers flexible downweighting or borrowing from source q depending on the value of $w_{q}$ . Briefly, the values of $a_{01},b_{01}$ are chosen such that the first Gamma mixture component has its mass on small values, therefore when $w_{q}\to 1$ , data from source q is increasingly discounted. At the extreme, setting $w_{q}=1$ indicates complete irrelevance of information from source q to the new trial. On the other hand, values of $a_{02},b_{02}$ are chosen such that the second Gamma mixture component has its mass on large values. In this case, setting $w_{q}\to 0$ results in a greater degree of incorporation of information from source q . Setting $w_{q}=0$ indicates exchangeability between $\lambda_{q}$ and $\mu_{\Delta }$ , that is $\mu_{\Delta (q)}∼N(\theta_{q},\tau_{q}^{2})$ . It is anticipated in a real application that, at the design stage of a new trial, $w_{q}\;\epsilon \;[0,1]$ are chosen in collaboration with a subject-matter expert(s) to reflect the anticipated degree of (ir)relevance between each historical trial and the new experiment. As detailed in Zheng et al.,^14^ the Gamma mixture prior in (4) can be approximated by matching the first two moments of a unimodal *t* mixture distribution. This leads to an approximation of the between-trial variance (i.e. between source q and the new experiment),

(5) $$\nu_{q}^{-1}≃\frac{w_{q}b_{01}}{a_{01}-1}+\frac{(1-w_{q})b_{02}}{a_{02}-1}.$$

We note that if we were being fully Bayesian we would keep the prior for $\nu_{q}$ in its distributional form, however in this paper we are looking to propose an asymptotically approximate sample size formula and so we make a simplifying assumption. The variance between each source q and the new trial is therefore estimated as

$$\xi_{q}^{2}=\tau_{q}^{2}+\frac{w_{q}b_{01}}{a_{01}-1}+\frac{(1-w_{q})b_{02}}{a_{02}-1}.$$

The above equation for $\xi_{q}^{2}$ highlights the importance of choosing values of $a_{01}$ , $b_{01}$ , $a_{02}$ , $b_{02}$ according to the minimum and maximum amount of information to borrow from external sources. We generally suggest choosing values of $a_{01}$ , $b_{01}$ so that the discounting term, $b_{01}/(a_{01}-1)$ , is large enough to effectively discount all information from a particular source when $w_{q}=1$ (i.e. so that $\xi_{q}^{2}\gg \tau_{q}^{2}$ when $w_{q}=1$ ). Similarly, $a_{02}$ , $b_{02}$ should be chosen so that the borrowing term, $b_{02}/(a_{02}-1)$ , is sufficiently small (i.e. close to zero) to enable all information to be incorporated from a particular source when $w_{q}=0$ (i.e. so that $\xi_{q}^{2}\to \tau_{q}^{2}$ when $w_{q}=0$ ). Alternatively, users may be interested in exploring various values for $a_{01},b_{01},a_{02},b_{02}$ to adjust the minimum/maximum sample size saving that is available. We encourage the end-user to use our openly available code to obtain this suited for their context.

### 2.3. Aggregating multiple distributions to form a collective prior

In Zheng et al.,^14^ an informative collective prior (hereafter referred to as ‘CP’) is formed by aggregating the Q predictive distributions in (3) into a single prior such that $\mu_{\Delta }|y_{1},\ldots ,y_{Q}∼N(\theta_{\mathrm{CP}},\sigma_{\mathrm{CP}}^{2})$ using the convolution operator for the sum of normal random variables,^16^ where

$$\theta_{\mathrm{CP}}=\sum_{q=1}^{Q}p_{q}\theta_{q},\;\sigma_{\mathrm{CP}}^{2}=\sum_{q=1}^{Q}p_{q}^{2}\xi_{q}^{2}.$$

$p_{q}$ are synthesis weights, set to a decreasing function of $w_{q}$ , such that are all between 0 and 1 and sum to 1 . In Zheng et al.,^14^

(6) $$p_{q}=\frac{\exp\;(-w_{q}^{2}/c_{0})}{\sum_{q=1}^{Q}\exp\;(-w_{q}^{2}/c_{0})},$$

where $c_{0}$ is a pre-defined concentration parameter which governs how much influence $w_{q}$ have on $p_{q}$ . Further details on the $p_{q}$ function in (6) and how to choose $c_{0}$ are provided in Zheng and Wason^17^ and Zheng et al.^14^ The CP is updated by the trial data to give the posterior,

$$\mu_{\Delta }|y_{1},\ldots ,y_{Q},y_{new}∼N\left(d_{\theta_{1}},\sigma_{\theta_{1}}^{2}\right),$$

In the same way as Equations (1) and (2), the posterior mean and variance are given by

$$d_{\theta_{1}}=\frac{\theta_{\mathrm{CP}}\cdot \sigma_{\mathrm{CP}}^{-2}+({\bar{y}}_{T}-{\bar{y}}_{C})\cdot nR(1-R)/\sigma_{0}^{2}}{\sigma_{\mathrm{CP}}^{-2}+nR(1-R)/\sigma_{0}^{2}}$$

and

$$\sigma_{\theta_{1}}^{2}={\left(\sigma_{\mathrm{CP}}^{-2}+\frac{nR(1-R)}{\sigma_{0}^{2}}\right)}^{-1}.$$

### 2.4. Varying $w_{q}$ to alter the amount of information from source q

For fixed $\tau_{q}^{2},a_{01},b_{01},a_{02},b_{02}$ , $c_{0}$ , the CP precision $\sigma_{\mathrm{CP}}^{-2}$ is a function of $w_{q}$ and is a measure of the amount of prior information on the treatment effect in the new trial (which varies depending on the values of $w_{q}$ ),

(7) $$\begin{array}{rl} \sigma_{\mathrm{CP}}^{-2} & ={\left[\sum_{q=1}^{Q}p_{q}^{2}\xi_{q}^{2}\right]}^{-1}\\ & ={\left[\sum_{q=1}^{Q}p_{q}^{2}\left(\tau_{q}^{2}+\frac{w_{q}b_{01}}{a_{01}-1}+\frac{(1-w_{q})b_{02}}{a_{02}-1}\right)\right]}^{-1}. \end{array}$$

**Figure 1.**
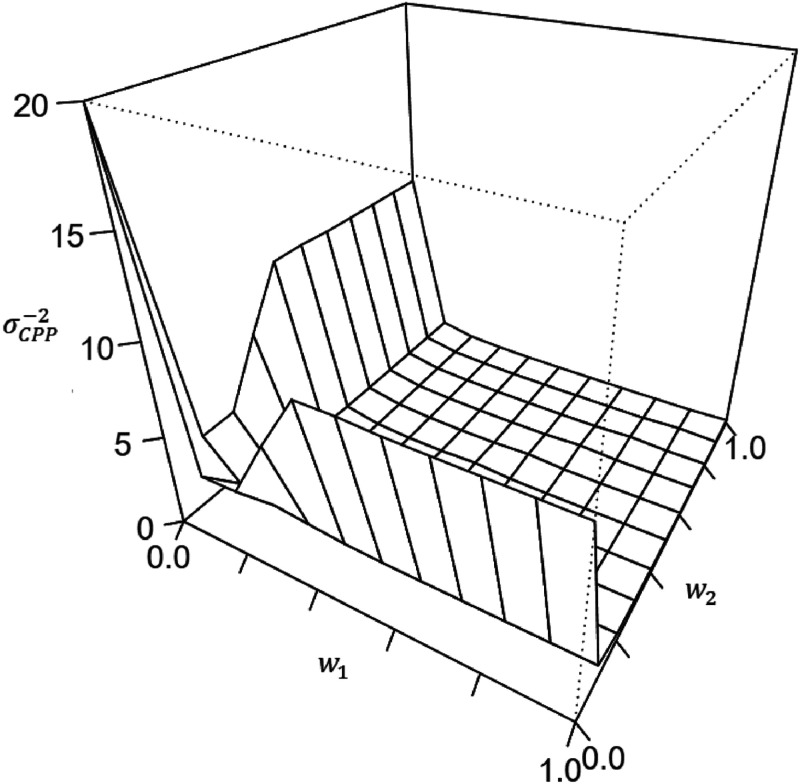
Collective prior (CP) precision, $\sigma_{\mathrm{CP}}^{-2}$ (equation 7), with respect to varying discrepancy weights, $w_{1}$ and $w_{2}$ , for borrowing from two historical sources, q=1,2 . Undesirable nonmonotonicity can clearly be seen around $w_{1}=0$ and $w_{2}=0$ .

In Figure 1, we visualize how $\sigma_{\mathrm{CP}}^{-2}$ varies according to $w_{q}$ in an example when Q=2 . For illustrative purposes, values of all other parameters are held fixed ( $\tau_{1}^{2}=\tau_{2}^{2}=0.1,a_{01}=1.1,b_{01}=1.1,a_{02}=1\times 10^{6},b_{02}=1,c_{0}=0.05$ ). It can be seen that for $w_{1}=w_{2}=0$ , corresponding to full incorporation of information from both historical sources, the CP precision is maximized (as desired). Similarly, for $w_{1}=w_{2}=1$ , corresponding to full discounting of information from both sources, the CP precision is minimized (as desired).

We nonetheless also see the (undesirable) highly nonlinear nature of $\sigma_{\mathrm{CP}}^{-2}$ with respect to varying $w_{q}$ in two respects. Firstly, it is clear that the majority of the change in prior precision occurs rapidly across $w_{q}$ , rather than evenly as we would like; beyond around $w_{q}>0.2$ , there is almost no discernible change in $\sigma_{\mathrm{CP}}^{-2}$ . Assuming that $w_{q}$ are expert elicited probabilities, this could result in a large loss of information because specifying any $w_{q}\;⪆\;0.2$ will result in almost full discounting of data from source q . This rapid nonlinear change is due to the functional form of the precision (specifically the general rectangular hyperbolic shape that results from taking the reciprocal of the variance), therefore occurs for any Q .

Secondly, and more importantly, when Q>1 , local minima/maxima can be seen around $w_{1}=0$ and $w_{2}=0$ . This nonmonotonic behaviour in equation (7) occurs whenever Q>1 due the method of prior aggregation as well as the higher order terms in $w_{q}$ introduced by the synthesis weighting function, equation (6). This is in contrast to how we would fundamentally wish the discrepancy weights to behave; it should be the case that increasing $w_{q}$ always leads to decreasing $\sigma_{\mathrm{CP}}^{-2}$ .

To be clear, this is a general problem, and not only for a specific set of parameters; that is nonlinearity (hyperbolic change and nonmonotonicity) of the prior precision occurs in varying degrees for any value of Q and regardless of the values that the other parameters are fixed at. These two issues mean that $w_{q}$ are not interpretable as probabilities and hinder communication with subject-matter experts to elicit sensible values at the trial design stage.

An alternative method of prior aggregation (and therefore a new way of formulating the CP precision) is necessary so that the nonlinearity has a simpler form. Specifically, the CP precision should be monotonically decreasing with respect to increasing $w_{q}$ . Details of our proposal are given in Section 3.1. Following derivation of a Bayesian sample size formula in Section 3.3, we also seek to recalibrate the weights. This is achieved in Section 3.4 via a functional transformation of each $w_{q}\to w_{q}^{\prime }$ , where $w_{q}^{\prime }=f(w_{q})$ , such that the prior precision (and therefore the derived sample size function) varies linearly with respect to $w_{q}\;\epsilon \;[0,1]$ .

## 3. Methods

### 3.1. Proposed method of prior aggregation

Following the set of predictive priors in (3), we propose an alternative method of prior aggregation suggested in Winkler.^18^ This results in a new CP, $\mu_{\Delta }|y_{1},\ldots ,y_{Q}∼N(\theta_{{\mathrm{CP}}^{*}},\sigma_{{\mathrm{CP}}^{*}}^{2})$ , where

$$\begin{array}{rl} \theta_{{\mathrm{CP}}^{*}} & =\sum_{q=1}^{Q}p_{q}^{*}\theta_{q},\;\sigma_{{\mathrm{CP}}^{*}}^{2}={\left(\sum_{q=1}^{Q}\xi_{q}^{-2}\right)}^{-1},\\ p_{q}^{*} & =\frac{\xi_{q}^{-2}}{\left(\sum_{q=1}^{Q}\xi_{q}^{-2}\right)}. \end{array}$$

As in Section 2.3, the CP mean, $\theta_{{\mathrm{CP}}^{*}}$ , is a weighted linear sum of the means from (3). Synthesis weights $p_{q}^{*}$ now incorporate information on both $\tau_{q}^{2}$ and $w_{q}$ , rather than only $w_{q}$ as in equation (6) (since $\xi_{q}^{-2}=(\tau_{q}^{2}+(w_{q}b_{01}/(a_{01}-1))+((1-w_{q})b_{02}/(a_{02}-1)){)}^{-1}$ ). This preserves the desirable property that smaller $w_{q}$ correspond to larger $p_{q}^{*}$ , and introduces the (also desirable) property that smaller $\tau_{q}^{2}$ correspond to larger $p_{q}^{*}$ . As required, $p_{q}^{*}$ sum to 1 and are all between 0 and 1 .

The CP variance, $\sigma_{{\mathrm{CP}}^{*}}^{2}$ , is the reciprocal of the sum of the precisions, $\xi_{q}^{-2}$ . Again, this preserves the desirable property that a smaller $w_{q}$ results in source q receiving a larger weight in $\sigma_{{\mathrm{CP}}^{*}}^{-2}$ . The formulation of the CP mean and variance in this manner is exactly in line with the theory of Bayesian updating of normal distributions with conjugate priors, with an initial noninformative prior for $\mu_{\Delta }$ (as discussed in Winkler^18^).

**Figure 2.**
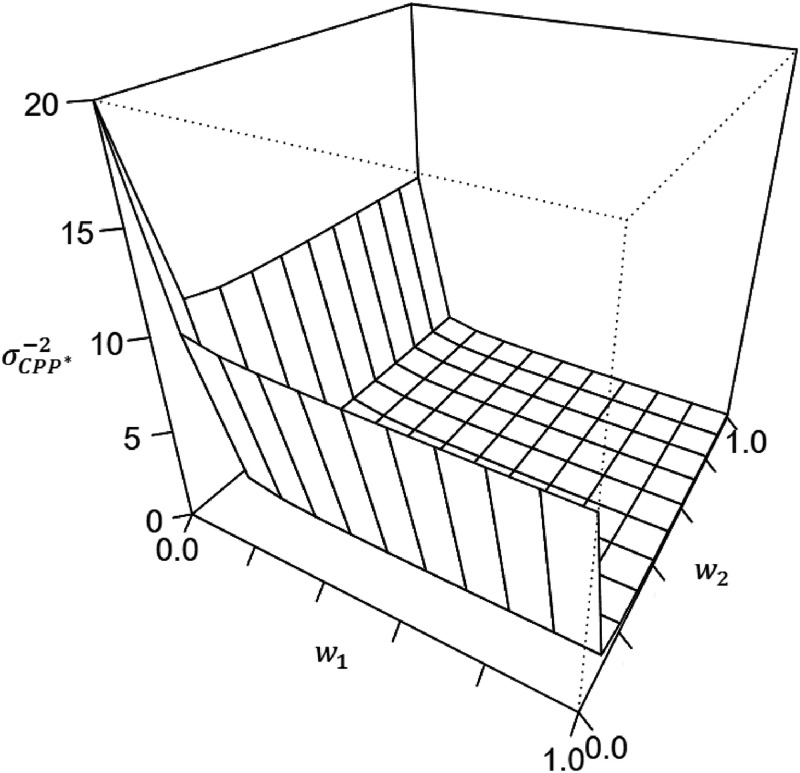
Proposed collective prior (CP) precision, $\sigma_{{\mathrm{CP}}^{*}}^{-2}$ (equation (8)), with respect to varying discrepancy weights, $w_{1}$ and $w_{2}$ , for borrowing from two historical sources, q=1,2 . The prior precision is now monotonically decreasing with respect to increasing $w_{q}$ .

An advantage of both the proposed prior aggregation method and the method detailed in Section 2.3 is that they allow for analytic sample size calculations. The proposed aggregation method preserves the desirable properties of the previous method of prior aggregation (described above) as well as fitting neatly into our Bayesian framework. However, central to the purpose of this paper, the nonlinearity of $\sigma_{{\mathrm{CP}}^{*}}^{-2}$ in equation (8) with respect to varying $w_{q}$ now has a simpler pattern when compared with equation (7). Crucially, the proposed CP variance, $\sigma_{{\mathrm{CP}}^{*}}^{2}$ , no longer relies on the original synthesis weights in equation (6), which caused the undesirable non-monotonic behaviour observed in Figure 1. This means that the precision, $\sigma_{{\mathrm{CP}}^{*}}^{-2}$ , is now strictly monotonically decreasing over $w_{q}$ . This can be proven by examining the first derivative of $\sigma_{{\mathrm{CP}}^{*}}^{-2}$ with respect to each $w_{q}$ which is always negative. In contrast to equation (7), this is now how we would wish $w_{q}$ to behave – that is increasing $w_{q}$ should always lead to decreased prior precision. This is visualized in Figure 2 using the same parameters as Figure 1 for borrowing from two historical datasets.

Additional motivation to use this method of aggregation in our particular application is that terms in the CP precision relating to each $w_{q}$ are now linearly independent of each other, that is

(8) $$\begin{array}{rl} \sigma_{{\mathrm{CP}}^{*}}^{-2} & =\sum_{q=1}^{Q}\xi_{q}^{-2}\\ & =\sum_{q=1}^{Q}\left[{\left(\tau_{q}^{2}+\frac{w_{q}b_{01}}{a_{01}-1}+\frac{(1-w_{q})b_{02}}{a_{02}-1}\right)}^{-1}\right]. \end{array}$$

This means we can now achieve linearization of $w_{q}$ with respect to sample size (details in the following sections), which would be impossible to achieve by the previous aggregation method due to the issue of nonmonotonicity. As in Section 2, the CP is updated by the trial data to give the posterior,

$$\mu_{\Delta }|y_{1},\ldots ,y_{Q},y_{new}∼N\left(d_{\theta^{*}},\sigma_{\theta^{*}}^{2}\right),$$

where

(9) $$d_{\theta^{*}}=\frac{\theta_{{\mathrm{CP}}^{*}}\cdot \sigma_{{\mathrm{CP}}^{*}}^{-2}+({\bar{y}}_{T}-{\bar{y}}_{C})\cdot nR(1-R)/\sigma_{0}^{2}}{\sigma_{{\mathrm{CP}}^{*}}^{-2}+nR(1-R)/\sigma_{0}^{2}}$$

and

(10) $$\sigma_{\theta^{*}}^{2}={\left(\sigma_{{\mathrm{CP}}^{*}}^{-2}+\frac{nR(1-R)}{\sigma_{0}^{2}}\right)}^{-1}.$$

### 3.2. Bayesian decision making framework

We now introduce a Bayesian decision making framework proposed in Whitehead et al.^19^ For pre-specified posterior decision thresholds η and ζ , we seek a sample size to guarantee we have sufficient evidence to conclude either efficacy or futility respectively. These thresholds represent the degree of evidence we would require to be convinced of efficacy or futility of treatment over control. Explicitly, if $P(\mu_{\Delta }>0)>\eta$ then we conclude that the treatment is efficacious and if $P(\mu_{\Delta }\leq \delta )>\zeta$ then we conclude that the treatment is futile, where η and ζϵ[0,1] and δ is some minimally clinically important treatment effect size.

For a generic posterior distribution $\mu_{\Delta }∼N(d_{\theta },\sigma_{\theta }^{2})$ , the probability that the treatment effect is greater than zero is

$$P(\mu_{\Delta }>0)=1-\Phi \left(-\frac{d_{\theta }}{\sigma_{\theta }}\right)=\Phi \left(\frac{d_{\theta }}{\sigma_{\theta }}\right),$$

where Φ(⋅) denotes the standard normal cumulative distribution function. Therefore, we will conclude convincing evidence of treatment benefit when $d_{\theta }/\sigma_{\theta }\geq z_{\eta }$ , where $z_{\eta }$ satisfies $\Phi (z_{\eta })=\eta$ .

Similarly, the posterior probability that the treatment effect is less than (or equal to) δ is

$$P(\mu_{\Delta }\leq \delta )=\Phi \left(\frac{\delta -d_{\theta }}{\sigma_{\theta }}\right).$$

Therefore, convincing evidence of treatment futility occurs when $(\delta -d_{\theta })/(\sigma_{\theta })\geq z_{\zeta }$ , where $z_{\zeta }$ satisfies $\Phi (z_{\zeta })=\zeta$ .

### 3.3. Bayesian sample size formula

Following the same approach detailed in Zheng et al.,^15^ to reach a decisive conclusion regarding treatment efficacy, we require a large enough sample size such that either $d_{\theta }/\sigma_{\theta }\geq z_{\eta }$ or $(\delta -d_{\theta })/\sigma_{\theta }\geq z_{\zeta }$ , that is

$$\frac{d_{\theta }}{\sigma_{\theta }}+\frac{\left(\delta -d_{\theta }\right)}{\sigma_{\theta }}\geq z_{\eta }+z_{\zeta }.$$

Simplifying and rearranging, this is equivalent to requiring that

(11) $$\frac{1}{\sigma_{\theta }^{2}}\geq {\left(\frac{z_{\eta }+z_{\zeta }}{\delta }\right)}^{2}.$$

We see that the left hand side of (11) is equal to the posterior precision. Replacing $\sigma_{\theta }^{2}$ with the variance in (2), we therefore obtain a Bayesian sample size formula in the case of no borrowing,

(12) $$n\geq \frac{\sigma_{0}^{2}}{R(1-R)}\left({\left(\frac{z_{\eta }+z_{\zeta }}{\delta }\right)}^{2}-\frac{1}{s_{0}^{2}}\right).$$

Note that if we wished to consider a purely frequentist formulation of the problem, then the necessary sample size is simply,

(13) $$n\geq \frac{\sigma_{0}^{2}}{R(1-R)}{\left(\frac{z_{1-\alpha }+z_{1-\beta }}{\delta }\right)}^{2},$$

where α and β are the usual parameters set to control type I and type II error rates, respectively.

Replacing $1/(s_{0}^{2})$ in (12) with $\sigma_{{\mathrm{CP}}^{*}}^{-2}$ from (8), we obtain our sample size calculation informed by Q sources of historical data,

$$n\geq \frac{\sigma_{0}^{2}}{R(1-R)}\left({\left(\frac{z_{\eta }+z_{\zeta }}{\delta }\right)}^{2}-\sigma_{{\mathrm{CP}}^{*}}^{-2}\right),$$

that is

(14) $$n\geq \frac{\sigma_{0}^{2}}{R(1-R)}\left({\left(\frac{z_{\eta }+z_{\zeta }}{\delta }\right)}^{2}-\sum_{q=1}^{Q}\left[{\left(\tau_{q}^{2}+\frac{w_{q}b_{01}}{a_{01}-1}+\frac{(1-w_{q})b_{02}}{a_{02}-1}\right)}^{-1}\right]\right),$$

with $w_{q}\;\epsilon \;[0,1],\;\;a_{01},a_{02}>1$ . We note explicitly the assumptions embedded into this sample size formula, which are common to many normal models. The validity of the sample size calculation depends on these assumptions being satisfied:

1. Common (and known) variance in outcomes from the new trial.
2. Independence of observations.
3. Homoscedasticity and normality of residuals.

For non-normal data, a suitably adapted formula based on the approach of constructing a normal test statistic in the generalized linear model framework via a transformation could be applied. In Supplemental Materials A.1, A2 and A.3, we demonstrate this by deriving sample size formulas for RCTs with binary and time-to-event data, and for single-arm settings with binary outcomes.

### 3.4. Interpretable discrepancy weights

We now detail the linearization steps which result in $w_{q}$ that are directly interpretable as a degree of discrepancy on the information scale, ϵ[0,1] . The idea is similar to the idea of functional uniform priors proposed in Bornkamp^20,21^ for nonlinear regression, in which a method for formulating a prior for a parameter of interest is proposed such that it is uniform in the space of functional shapes of the underlying nonlinear function. We start by isolating each nonlinear part of the sample size function in equation (14) with respect to $w_{q}$ (for fixed $\tau_{q}^{2},a_{01},b_{01},a_{02},b_{02}$ ). These are the individual precision terms making up $\sigma_{{\mathrm{CP}}^{*}}^{-2}$ in equation (8), that is

(15) $$\xi_{q}^{-2}(w_{q})={\left(\tau_{q}^{2}+\frac{w_{q}b_{01}}{a_{01}-1}+\frac{(1-w_{q})b_{02}}{a_{02}-1}\right)}^{-1}.$$

Step 1: Perform linear interpolation on (15):

(16) $$h(w_{q})=(1-w_{q})\xi_{q}^{-2}(0)+w_{q}\xi_{q}^{-2}(1)=\xi_{q}^{-2}(0)+w_{q}(\xi_{q}^{-2}(1)-\xi_{q}^{-2}(0)).$$

This essentially ‘draws a line’ between $\xi_{q}^{-2}(w_{q}=0)$ and $\xi_{q}^{-2}(w_{q}=1)$ so that changes in $\xi_{q}^{-2}$ (and therefore the corresponding sample size) are spread evenly across the full range of $w_{q}\;\epsilon \;[0,1]$ . This also necessarily ensures that the mapping $w_{q}\to w_{q}^{\prime }$ preserves the property that $w_{q}=0\to w_{q}^{\prime }=0$ and $w_{q}=1\to w_{q}^{\prime }=1$ .

Step 2: Find the inverse of (15). This allows calculation of any $w_{q}$ value corresponding to a given $\xi_{q}^{-2}$ :

(17) $$g(\xi_{q}^{-2})=(\xi_{q}^{-2}{)}^{\left(-1\right)}$$

Step 3: Substitute the linearized $\xi_{q}^{-2}$ values obtained in (16) into (17):

(18) $$f(w_{q})=g(h(w_{q}))=w_{q}^{\prime },$$

$f(w_{q})$ is now the necessary transformation of $w_{q}\to w_{q}^{\prime }$ . Now, if we obtain expert elicited values of $w_{q}$ , corresponding to a percentage degree of discrepancy between each historical source and the new trial, we can use equation (14) with $w_{q}^{\prime }$ to incorporate $((1-w_{q})\times 100)\%$ of the information from the corresponding historical dataset in the planning of the new trial.

This transformation is possible due to the proposed method of prior aggregation. Unlike the original $\sigma_{\mathrm{CP}}^{-2}$ in equation (7), the proposed $\sigma_{{\mathrm{CP}}^{*}}^{-2}$ is a monotonic function in $w_{q}$ , and each of the $\xi_{q}^{-2}$ terms (which form $\sigma_{{\mathrm{CP}}^{*}}^{-2}$ ) are linearly independent (i.e. separated by the addition operator). The transformation procedure can easily be extended to any number of sources, q=1,…,Q , with each functional transformation of $w_{q}\to f(w_{q})=w_{q}^{\prime }$ being performed independently with no additional complexity.

**Figure 3.**
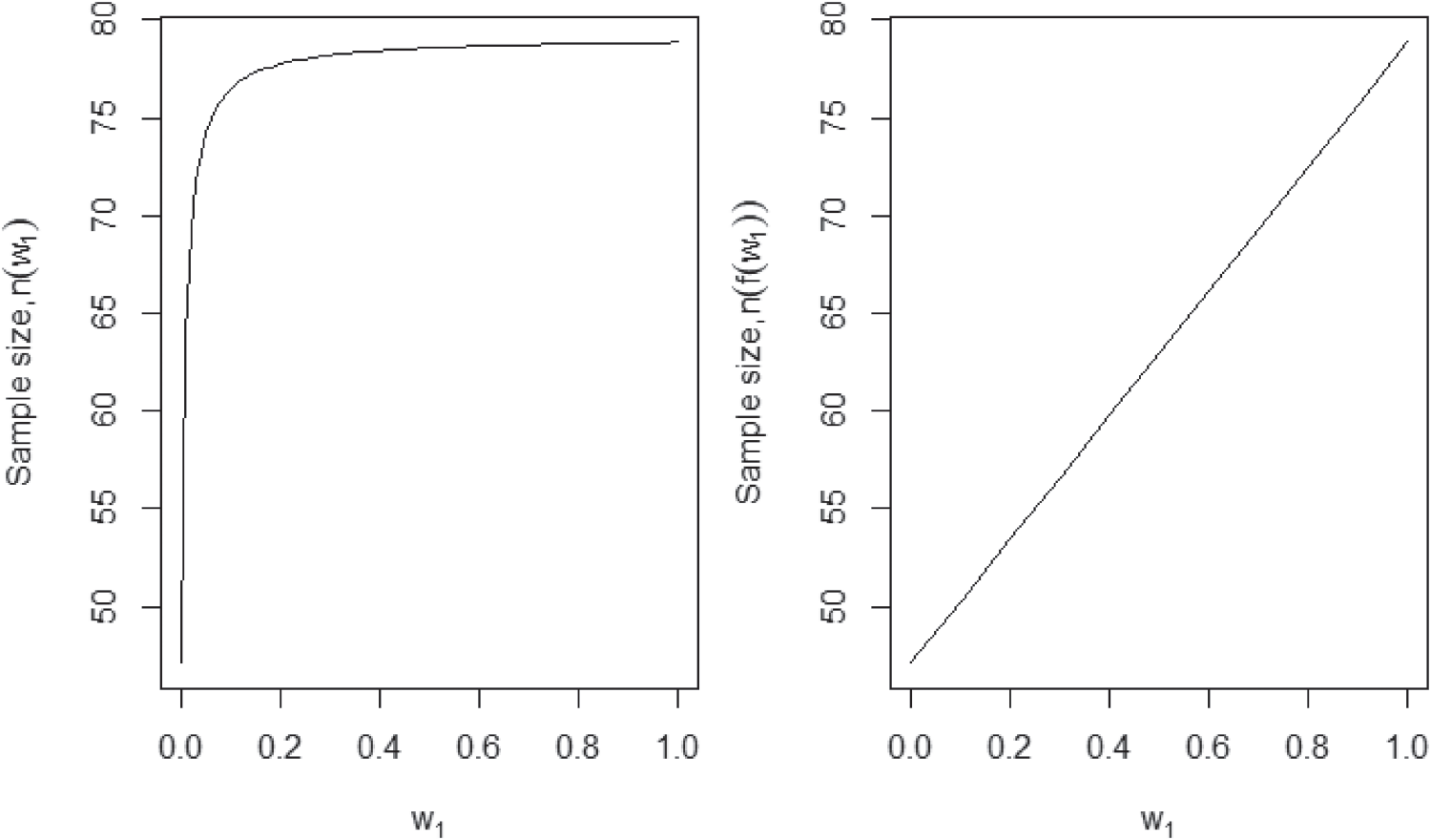
Sample size, n , with respect to varying $w_{1}$ , for borrowing from a single source of data, both before (left) and after (right) functional transformation of $w_{1}\to f(w_{1})=w_{1}^{\prime }$ . Note that as required, minimum and maximum sample sizes corresponding to $w_{1}=0$ and $w_{1}=1$ respectively remain identical in both cases.

**Figure 4.**
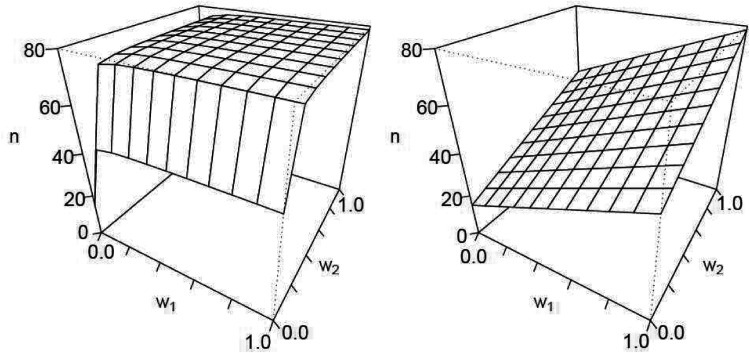
Sample size n (vertical axis) borrowing from two historical sources, with respect to varying $w_{1}$ and $w_{2}$ . The left figure is before the functional transformation of $w_{q}\to w_{q}^{\prime }$ , that is $n(w_{q},\tau_{q}^{2})$ , the right figure is after, that is $n(w_{q}^{\prime },\tau_{q}^{2})$ .

The effect is visualized in Figures 3 and 4, which compare the sample size function (plotted at the boundary of the inequality, that is the smallest possible sample size fulfilling equation (14)) with respect to $w_{q}$ before and after the functional transformation of $w_{q}\to f(w_{q})=w_{q}^{\prime }$ . These examples are for the simplest cases of borrowing from one and two historical datasets.

Sample sizes corresponding to $n(w_{1}=0,w_{2}=0),n(w_{1}=1,w_{2}=0),n(w_{1}=0,w_{2}=1)$ and $n(w_{1}=1,w_{1}=1)$ remain fixed after the transformation of $w_{q}\to w_{q}^{\prime }$ as required. This will be the case for borrowing from any number of sources, that is borrowing from Q sources will have $2^{Q}$ fixed points corresponding to each unique combination of $w_{1}=\{0,1\},\ldots ,w_{Q}=\{0,1\}$ . Between these fixed points, via the proposed transformation, the change in the sample size is evenly distributed across $w_{1},\ldots ,w_{Q}\;\epsilon \;[0,1]$ . As before, the sample size is minimized with full incorporation of information from all sources, that is $\{w_{1},\ldots ,w_{Q}\}=0$ and maximized with full discounting of information from both sources, that is $\{w_{1},\ldots ,w_{Q}\}=1$ .

## 4. Application to the design of a randomized controlled trial in Alzheimer’s disease

In this section, we consider how the proposed method could be applied to determine an appropriate sample size for a hypothetical new trial using real data from several relevant historical RCTs.

Alzheimer’s disease (AD) is a chronic age-related illness characterized by cognitive decline. It is the most common form of dementia, with incidence increasing globally due to increasing life expectancy. There are limited pharmaceutical interventions which are effective in reducing symptoms of cognitive decline, however a systematic review by Du et al.^22^ highlighted that several previous studies have suggested that exercise may slow the progression of cognitive decline in patients with AD.

Consider planning a new two-arm RCT to investigate whether physical activity can improve cognition in patients with Alzheimer’s disease. The two treatments to be compared in the new trial are denoted T (physical activity) and C (standard/usual care). The primary outcome is the difference in treatment group means at a single post-randomization followup timepoint in the Mini Mental State Examination (MMSE) score.^23^ MMSE is a 30-point questionnaire that provides a summary measure of cognitive function where a higher score represents better cognitive performance. It is used extensively in clinical research settings to estimate the severity of impairment, and to document change in impairment over time. Suppose in the new trial that the MMSE of each subject at 4 months post-randomization will be denoted by $y_{ij},i=1,\ldots ,n_{j},j=T,C$ , and $y_{i,j}$ will be treated as normally distributed with mean $\mu_{j}$ and common (known) variance $\sigma_{0}^{2}$ , as in Section 3.3. The observed difference in means ${\bar{Y}}_{T}-{\bar{Y}}_{C}={\bar{Y}}_{\Delta }$ is assumed to be normally distributed, ${\bar{Y}}_{\Delta }∼N(\mu_{\Delta },\sigma_{0}^{2}/nR(1-R))$ with positive values indicating an advantage for the physical activity group. Based on a recent study of MMSE scores in those with cognitive impairments, $\sigma_{0}^{2}=3.69^{2}$ .^24^

Consider first a frequentist formulation of the sample size calculation. Suppose we wish to detect a minimum clinically important difference (MCID) between treatment groups of δ=1 point on the MMSE (it was reported by Mishra et al.^25^ that MCID thresholds for MMSE in AD trials are commonly between 1 and 3 points). For a one-sided type I error rate α=0.05 and power 1−β=0.80 , the total sample size required is minimized by equal allocation to treatment and control groups, that is, R=0.5 . For these parameters, equation (13) yields a total sample size of n=338 (rounded up to the nearest even integer). The Bayesian sample size calculation with no borrowing, equation (12), gives the same result setting a large $s_{0}^{2}$ (e.g. $s_{0}^{2}=100$ ), with η=0.95 and ζ=0.80 .

For obvious reasons, recruiting large numbers of patients onto AD trials might be challenging, with limitations due to ethical and practical issues. Furthermore, high costs can be a concern with trial participants necessarily needing more intense monitoring compared to cognitively intact individuals.^26^

Now, suppose that data from 7 historical trials is available with which to form an informative prior for $\mu_{\Delta }$ , summarized in Table 1.

**Table 1.** Results of seven historical RCTs measuring MMSE outcomes for individuals with AD, adapted from Du et al.^22^

		Experimental			Control			Difference	
q	Study	Mean	SD	$n_{T}$	Mean	SD	$n_{C}$	$\theta_{q}$	$\tau_{q}^{2}$
1	Vreugdenhil et al.^27^	23.9	5	20	19	7.7	20	4.90	4.21
2	Hoffmann et al.^28^	23.9	3.4	107	23.9	3.9	93	0	0.27
3	Venturelli et al.^29^	12	2	11	6	2	10	6	0.76
4	Dky et al.^30^	17.4	5.7	24	19.2	4.2	28	-1.8	1.89
5	Yang et al.^31^	22.83	2.75	25	19.54	3.43	25	3.29	0.77
6	Holthoff et al.^32^	22.11	0.57	15	20.72	0.55	15	1.39	0.04
7	Kwak et al.^33^	19.1	6.5	15	12.3	6.7	15	6.8	5.81

Note: Treatment effects have been summarized in the form of $\lambda_{q}|y_{q}∼N(\theta_{q},\tau_{q}^{2})$ . RCT: randomized controlled trial; MMSE: mini mental state examination; AD: Alzheimer’s disease.

It is clear from Table 1 that there is substantial heterogeneity between studies, therefore with the help of a clinical expert we suppose we have elicited probabilities $w_{1},\ldots ,w_{Q}\;\epsilon \;[0,1]$ which quantify the irrelevance of each historical trial in respect of the new study.

We note that it might be easier to elicit these quantities as a degree of relevance (rather than degree of skepticism); for example, if an expert thinks data source q is 25% relevant to the new trial then we set $w_{q}=0.75$ . We also note that the proposed methodology assumes that a single expert is consulted, or that multiple experts can agree on single values for $w_{q}$ . The process of eliciting and reconciling multiple expert opinions on probabilities is a complex topic outside the scope of this paper; see, for example, Hora^34^ for an in depth discussion. For illustrative purposes, let us assume that we have elicited a set of probabilities $w_{q}\;\epsilon \;[0,1]$ , with $w_{1}=0.65,w_{2}=0.90,w_{3}=0.75,w_{4}=0.75,w_{5}=0.40,w_{6}=0.95,w_{7}=0.50$ . This set would imply a desire to incorporate the most amount of information from source 5 and least from source 6 .

Firstly, using equation (18) to transform $w_{q}\to w_{q}^{\prime }$ results in $w_{1}^{\prime }=7.66\times 10^{-3},w_{2}^{\prime }=2.37\times 10^{-3},w_{3}^{\prime }=2.26\times 10^{-3},w_{4}^{\prime }=5.58\times 10^{-3},w_{5}^{\prime }=5.10\times 10^{-4},w_{6}^{\prime }=7.86\times 10^{-4},w_{7}^{\prime }=5.69\times 10^{-3}$ . By the method in Section 3.1, using $w_{q}^{\prime }$ , $\theta_{q}$ and $\tau_{q}^{2}$ leads to an informative prior for the treatment effect in the new trial, $\mu_{\Delta }|y_{1},\ldots ,y_{7}∼N(\theta_{{\mathrm{CP}}^{*}},\sigma_{{\mathrm{CP}}^{*}}^{2})$ , where $\theta_{{\mathrm{CP}}^{*}}=2.33$ and $\sigma_{{\mathrm{CP}}^{*}}^{2}=0.34$ . equation (14) (setting $a_{01}=1.01,b_{01}=1.01,a_{02}=1\times 10^{6},b_{02}=1$ ), gives the total sample size (for η=0.95 and ζ=0.80 ) as n=176 (rounded up to the nearest even integer).

Note, that if we just used the ‘raw’ $w_{q}$ in equation (14), we would be faced with the issue of over-discounting (described in previous sections), resulting in a sample size of 332 . Also note that if we wished to include information to the specified degree (without transforming $w_{q}$ ), then we would have to have elicited values of $w_{1}=7.66\times 10^{-3},w_{2}=2.37\times 10^{-3},w_{3}=2.26\times 10^{-3},w_{4}=5.58\times 10^{-3},w_{5}=5.10\times 10^{-4},w_{6}=7.86\times 10^{-4},w_{7}=5.69\times 10^{-3}$ , which would have been very difficult to elicit even if the expert(s) had substantial statistical knowledge.

## 5. Performance evaluation

We present a brief simulation study where the purpose is to verify that the proposed sample size function and linearization technique achieve the pre-specified statistical properties across a range of scenarios. To be clear, according to the criteria of the Bayesian decision framework in Section 3.2, the sample size should be large enough to guarantee a conclusion of efficacy, such that P(θ>0)≥η , or, if not, futility, such that P(θ≤δ)≥ζ . The goal of the simulation study therefore is not to compare our sample size formula and linearization technique against another method, but rather to test the hypothesis that by the proposed method 100% of trials will reach a definitive conclusion.

### 5.1. Basic settings

Four contrasting configurations, A,…,D , of hypothetical historical data are investigated, with each containing historical information from 5 independent sources shown in Table 2. We suppose that probabilities $w_{q}=w_{1},\ldots ,w_{5}$ have been elicited to implement the proposed approach for borrowing information from each respective source. We fix $w_{q}$ to be the same across all four configurations to facilitate easier comparisons: $w_{1}=0.2,w_{2}=0.4,w_{3}=0.8,w_{4}=0.6,w_{5}=0.7$ . We suppose for demonstration purposes that data from historical data source 1 is considered particularly relevant to the new trial, setting $w_{1}=0.2$ (in a real situation, this might be for example because the historic trial has been performed most recently, or that it was undertaken at an earlier stage in the same pharmaceutical development pipeline). Sources 2–5 have been considered less relevant with $w_{2},\ldots ,w_{5}$ set accordingly.

**Table 2.** Configurations A,…,D , of hypothetical historical data where mean treatment effect parameter from source q is assumed to have been independently summarized by $\lambda_{q}∼N(\theta_{q},\tau_{q}^{2})$ .

			Historical data source, q :				
Config.	Config. description	Parameter	1	2	3	4	5
	Weak	$\theta_{q}$	0.10	0.24	0.37	0	-0.05
A	historical	$\tau_{q}^{2}$	1.25	0.73	0.92	1.29	0.66
	info.	$w_{q}$	0.20	0.40	0.80	0.60	0.70
		$\theta_{q}$	0	-0.05	2.14	0.37	1.10
B	Mixed 1	$\tau_{q}^{2}$	1.29	0.66	0.50	0.92	0.75
		$w_{q}$	0.20	0.40	0.80	0.60	0.70
		$\theta_{q}$	1.10	0.37	-0.05	2.14	0
C	Mixed 2	$\tau_{q}^{2}$	0.75	0.92	0.66	0.50	1.29
		$w_{q}$	0.20	0.40	0.80	0.60	0.70
	Strong	$\theta_{q}$	1.10	2.14	1.07	0.60	0.85
D	historical	$\tau_{q}^{2}$	0.75	0.50	0.82	0.89	0.26
	info.	$w_{q}$	0.20	0.40	0.80	0.60	0.70

Note: Each source is accompanied by a $w_{q}$ for borrowing of information, summarizing pre-experimental information about $\mu_{\Delta }$ .

Configuration descriptions classify the nature of the treatment effects observed in the historical trials, with ‘weak historical info’. meaning low/neutral relative treatment effects observed historically with relatively high variances, and ‘strong historical info’. indicating more positive historic treatment effects with comparatively smaller variances. Mixed 1 and Mixed 2 use a combination of $\theta_{q}$ and $\tau_{q}^{2}$ from A and D. Weights in Mixed 1 favour the neutral trials, while weights in Mixed 2 favour the more positive trials. Based on the example in Section 4, the MCID between treatment arms in the new trial is set to be δ=1 and we assume a common (known) variance in outcome measures of $\sigma_{0}^{2}=3.69^{2}$ . Probability boundaries for decision making in terms of efficacy are η=0.95 , and for futility, ζ=0.80 . For each configuration of historical data, a sample size is calculated for the new trial: First, using equation (18) to transform $w_{q}\to w_{q}^{\prime }$ , and then equation (14), with $w_{q}^{\prime }$ and $\tau_{q}^{2}$ (setting $a_{01}=1.01,b_{01}=1.01,a_{02}=1\times 10^{6},b_{02}=1$ ). Note that, although η and ζ have been set to be equivalent to the often used (1−α) and (1−β) in the frequentist paradigm (which are set to control type I error rate and power respectively), it must be remembered that these values do not represent the same quantity. As discussed in Whitehead et al.,^19^ there is no reason to assume any form of equivalence since their meanings are fundamentally different.

For the new trial, we set equal allocation to treatment and control, R=0.5 . Outcomes in the control group are generated for each configuration according to $Y_{iC}∼N(0,\sigma_{0}^{2}),i=1,\ldots n_{k}/2$ (where k=1,…,4 indexes configurations A,…,D ). Outcomes in the treatment group are generated according to $Y_{iT}∼N(\mu_{\Delta },\sigma_{0}^{2}),i=1,\ldots n_{k}/2$ . For each simulation replicate, true treatment effects are set to be one of the following:

1. Treatment efficacy, $\mu_{\Delta }=1$ .
2. Treatment futility, $\mu_{\Delta }=0$ .

A Bayesian analysis model is applied to each simulation replicate, with prior set according to the CP from each configuration. Evidence of treatment efficacy will be concluded if $P(\mu_{\Delta }>0)\geq 0.95$ . If $P(\mu_{\Delta }>0)<0.95$ then, according to our pre-specified criteria, it should be the case that $P(\mu_{\Delta }\leq \delta )\geq 0.80$ . Results are summarized for $\mu_{\Delta }=1$ and $\mu_{\Delta }=0$ , respectively by calculating the percentage of trials in which a decisive conclusion can be reached by averaging across 10,000 simulated trial replicates. This results in a total of 8 scenarios. The Bayesian analysis model is fitted analytically usingequations (9) and (10) in R version 4.2.1 (2022-06-23).

### 5.2. Results

Table 3 gives transformed values of $w_{q}\to w_{q}^{\prime }$ via the method described in Section 3.4.

**Table 3.** Transformed values of $w_{q}\to w_{q}^{\prime }$ .

Config.	$w_{q}$	0.20	0.40	0.80	0.60	0.70
A	$w_{q}^{\prime }$	$3.05\times 10^{-3}$	$4.76\times 10^{-3}$	$3.48\times 10^{-2}$	$1.86\times 10^{-2}$	$1.49\times 10^{-2}$
B	$w_{q}^{\prime }$	$3.14\times 10^{-3}$	$4.31\times 10^{-3}$	$1.93\times 10^{-2}$	$1.34\times 10^{-2}$	$1.69\times 10^{-2}$
C	$w_{q}^{\prime }$	$1.84\times 10^{-3}$	$5.98\times 10^{-3}$	$2.53\times 10^{-2}$	$7.33\times 10^{-3}$	$2.86\times 10^{-2}$
D	$w_{q}^{\prime }$	$1.84\times 10^{-3}$	$3.27\times 10^{-3}$	$3.12\times 10^{-2}$	$1.29\times 10^{-2}$	$5.96\times 10^{-3}$

CP: collective prior.

Table 4 displays sample sizes (rounded up to the nearest even integer to allow for equal allocation between treatment groups) for each configuration calculated using equation (14) with $w_{q}^{\prime }$ and $\tau_{q}^{2}$ , along with the corresponding prior parameters used for design and analysis. The prior for configuration A is centered closer to zero with a higher variance than the priors for other configurations, resulting in a sample size of n≥204 . The prior for configuration D is the most ‘enthusiastic’, centered on a positive treatment effect with a lower variance, resulting in n≥112 . Configuration B results in a prior centered on a low treatment effect, whereas the prior derived from configuration C is centered on a positive treatment effect. Configurations B and C result in priors with similar variances.

**Table 4.** Priors for treatment effect in the new experiment, $\mu_{\Delta }∼N(\theta_{{\mathrm{CP}}^{*}},\sigma_{{\mathrm{CP}}^{*}}^{2})$ , along with corresponding sample sizes (rounded up to nearest even integer for R=0.5 ) for configurations A,…,D .

Config.	$\theta_{{\mathrm{CP}}^{*}}(w_{q}^{\prime },\theta_{q},\tau_{q}^{2})$	$\sigma_{{\mathrm{CP}}^{*}}^{2}(w_{q}^{\prime },\tau_{q}^{2})$	n
A	0.131	0.405	204
B	0.515	0.358	186
C	1.015	0.325	170
D	1.276	0.242	112

CP: collective prior

Table 5 displays the percentage of simulated trials concluding that experimental treatment is efficacious (% Eff.) or futile (% Fut.) for each configuration in scenarios where $\mu_{\Delta }=1$ and $\mu_{\Delta }=0$ respectively. The percentage efficacious is defined as the percentage of trials out of 10,000 simulations in which $P(\mu_{\Delta }>0)\geq 0.95$ , while the percentage futile is the percentage of trials out of 10,000 simulations in which $P(\mu_{\Delta }>0)<0.95$ and $P(\mu_{\Delta }\leq 0.4)\geq 0.80$ . The total percentage is 100% in all scenarios, demonstrating that the pre-specified statistical properties are upheld by the proposed method.

**Table 5.** Percentage of simulated trials that conclude treatment is efficacious or futile when $\mu_{\Delta }=1$ and $\mu_{\Delta }=0$ (analyzed using informative priors for $\mu_{\Delta }$ as specified in Table 4.)

		$\mu_{\Delta }=1$ :			$\mu_{\Delta }=0$ :		
Config.	n	% Eff.	% Fut.	Total %	% Eff.	% Fut.	Total %
A	204	49.3	50.7	100	2.6	97.4	100
B	186	66.0	34.0	100	7.3	92.7	100
C	170	88.7	11.3	100	29.2	70.8	100
D	112	98.7	1.3	100	79.8	20.2	100

We emphasize that an investigation of frequentist operating characteristics was not the purpose of this section. Nonetheless, as anticipated, and as mentioned in Section 1, it is clear from Table 5 that to realize the benefits of historical borrowing (at least, in traditional frequentist terms), the treatment effect in the new trial should be similar to the treatment effect in historical trials. When this is the case, we observe higher ‘power’ (as in configurations C and D when $\mu_{\Delta }=1$ ) (or a lower ‘type I error rate’, as in configurations A and B when $\mu_{\Delta }=0$ ) by borrowing of information. However, this necessarily comes at the risk of a higher type I error rate/reduced power when there is a high degree of heterogeneity between historical and current trials. As discussed in Kopp-Schneider et al.,^35^ if one wishes to control the type I error rate in the traditional sense, all prior information must be disregarded in the analysis. It may however be desirable to determine the weight parameters alongside consideration of the type I error rate, which is agreed upon by sponsors and regulators during the design stage, as discussed, for example, in Lee.^36^ Whichever operating characteristics are considered, in any practical application, careful selection of historical trials for inclusion as well as extensive simulations at the trial design stage would be necessary.

## 6. Discussion

The central goal in this paper has been twofold: firstly, to offer a solution for the problem of nonmonotonic behaviour of discrepancy weights caused by the prior aggregation method proposed in Zheng et al.^14,15^ Our proposed alternative ensures that discrepancy weights behave monotonically with respect to the amount of information included from a particular source. This leads us to derive a Bayesian sample size formula and achieve our second goal of linearization to improve interpretability. Following our methodology, given a set of historical data sources, clinical expert(s) only have to specify the amount of information to borrow (discount) from each historic data source with respect to the current trial ( $w_{q}$ ) for a trial statistician to then to incorporate the specified amount of information (using $w_{q}^{\prime }$ ). We hope that these ideas can encourage effective communication between statisticians and subject-matter experts to elicit sensible values for these weights.

Focus in this work is on the design of a two-armed trial where there is prior information on the difference in means between treatment and control arms. We acknowledge that it is much more common in practice to consider borrowing only on the control arm (i.e. using historical control information to augment or replace a concurrent control). The methods presented here could be adapted to this case such that a prior would be formed for the arm-based statistic(s). In this case, the weight(s) would then relate to the anticipated (dis)similarity between the historical control data and the new control data, implying a reduced number of patients on the new control. As noted in Zheng et al.,^14^ selection of historical data on a single arm should be done carefully to avoid bias that may affect the inference of the difference in means.

There are a number of ways in which this work could be extended/generalized. One possibility would be extension to other Bayesian methods proposed for clinical trials which utilize weights for borrowing, such as the robust MAP prior.^6^ More broadly, the methodology could be applied in any research area (not just clinical trials) where it would be desirable to design an experiment using information from previous studies or external data.

In the case of applying the method to survival data (see Supplemental Materials), the assumption of an exponential distribution is analytically convenient, which is not uncommon especially in the practice of designing clinical trials. It is important to state that sample size formulae (including ours), based on assumptions for analytical convenience, are typically a good design approximation, but not an exact one. As a reviewer rightly noted, analysis of real survival data rarely relies on such assumptions. If knowing the exact analysis model to apply, a simulation-based approach to sample size determination would be more accurate.

We note that in this work we have assumed independence of historical data sources as a simplified case of aggregating information by the method of Winkler,^18^ in which a method is proposed in the case that historical sources are dependent. When historical studies are conducted on distinct patients the independence assumption would seem reasonable. However, if the historical data relate to multiple trials in the same patients (for example, phase II/III trials), then the dependence between studies could easily be accounted for by the same method in Winkler,^18^ with calculation of the pairwise correlations between sources.

Our sample size formula and linearization technique could also be extended to other clinical trial designs where borrowing can be incorporated; for example, combined phase II/III trials using borrowing from the phase II part of the trial to reduce the sample size for the phase III part, or a basket trial setting (for concurrent borrowing between subtrials) in which a sample size is sought for each subtrial, k=1,…,K , with sample sizes being solved as a system of K simultaneous equations.

The proposed methodology utilizes a single prior for both the design and analysis of the new experiment. There may be instances where it is desirable to modify the analysis prior according to the observed similarity between the historic datasets and current trial. In this case, a distributional distance metric ϵ[0,1] such as the Hellinger distance^37^ might be useful in updating $w_{q}$ for the analysis. However, as noted in Zheng et al.,^15^ this would affect the properties of the Bayesian decision making framework on which the sample size formula is based. Specifically, when $w_{q}$ are set to larger values in the analysis than in the design (i.e. less borrowing is implemented than planned), it may not be possible to reach a decisive conclusion regarding efficacy or futility. Conversely, using smaller $w_{q}$ in the analysis than the design (i.e. more borrowing is implemented than planned) would lead to a more precise posterior distribution which may have a higher risk of bias.

In our approach, we have restricted focus to known variance in outcome measure, $\sigma_{0}^{2}$ (common in many settings), and we approximated $\nu_{q}^{-1}$ by making some simplifying assumptions, which resulted in a closed form for the sample size calculation. One avenue for development would be a more fully Bayesian approach in which priors are specified for $\sigma_{0}$ and/or $\nu_{q}$ . Furthermore, in this paper we have focussed on the Bayesian decision making framework proposed in Whitehead et al.,^19^ however, it would be simple to adapt the sample size formula for consideration of other Bayesian properties. For example, a sample size formula controlling average properties of posterior interval probabilities could be achieved in a similar manner as in Zheng et al.,^14^ where a sample size formula is proposed for control of the average coverage criterion or the average length criterion; for implementation of our method this would simply require replacing the prior precision ( $\sigma_{\mathrm{CP}}^{-2}$ ) proposed in Zheng et al.^14^ with our alternative proposal ( $\sigma_{{\mathrm{CP}}^{*}}^{-2}$ ).

In conclusion, historical data from a range of sources are often available in the planning of a new trial, but inclusion of such data for study design and analysis is not common practice. Part of the reason might be difficulty in interpretability of discrepancy parameters. We hope our work will help to bridge this gap and encourage uptake of these innovative methods, however we caution that consideration of sample size on its own should not be the only factor when determining whether a borrowing method is appropriate. Simulation is generally still needed to evaluate its performance (bias, power, type I error, etc.).

## Supplemental Material

sj-pdf-1-smm-10.1177_09622802261432816 - Supplemental material for Bayesian sample size determination using robust commensurate priors with interpretable discrepancy weights Supplemental material, sj-pdf-1-smm-10.1177_09622802261432816 for Bayesian sample size determination using robust commensurate priors with interpretable discrepancy weights by Lou E Whitehead, James MS Wason, Oliver Sailer and Haiyan Zheng in Statistical Methods in Medical Research
