# Supplementary material for: Bayesian sample size determination using robust commensurate priors with interpretable discrepancy weights
Source: Stat Methods Med Res. 2026 Apr 16;35(5):1046–62. doi: 10.1177/09622802261432816 (PMC13272840; doi:10.1177/09622802261432816)
Supplement: sj-pdf-1-smm-10.1177_09622802261432816 - Supplemental material for Bayesian sample size determination using robust commensurate priors with interpretable discrepancy weights [file sj-pdf-1-smm-10.1177_09622802261432816.pdf]

Bayesian sample size determination using robust commensurate priors with interpretable discrepancy weights: **Supplementary Materials**

A Supplementary Materials

A.1 Bayesian sample size formula for a two-armed RCT with binary endpoints

Consider planning a new RCT to compare response rates between a treatment and control group using relevant historical data. We follow similar steps detailed in the Supplementary Material in Zheng et al. (2023), with two important differences:

- The sample size calculation here is for a two-arm RCT with borrowing from historical data, rather than a basket trial with multiple substudies borrowing from concurrent data.
- Our new formulation of $\sigma_{CP^*}^{-2}$, i.e.,

$$\begin{aligned}\sigma_{CP^*}^{-2} &= \sum_{q=1}^{Q} \xi_q^{-2} \\ &= \sum_{q=1}^{Q} \left[\left(\tau_q^2 + \frac{w_q b_{01}}{a_{01} - 1} + \frac{(1 - w_q) b_{02}}{a_{02} - 1} \right)^{-1} \right], \end{aligned} \tag{1}$$

  which is detailed in Section 3.1 of the main paper.

In the new trial, the observed proportion of responders in each arm of the sample is

$$\hat{\rho}_j = \sum_{i=1}^{n_j} Y_{ij}/n_j$$

where Y_{ij} is a binary indicator denoting response ($= 1$) or non-response ($= 0$) for subject $i = 1, ..., n_j$ in arm $j = T, C$. By the central limit theorem, and as long as the proportions are not close to 0 or 1, the log odds ratio of the sample

$$\log(\hat{OR}) = \log\left(\frac{\hat{\rho}_T(1 - \hat{\rho}_C)}{\hat{\rho}_C(1 - \hat{\rho}_T)}\right)$$

would be approximately normally distributed (Agresti, 2003),

$$\log(\hat{OR}) \dot{\sim} \mathcal{N}\left(\log\left(\frac{\rho_T(1-\rho_C)}{\rho_C(1-\rho_T)}\right), \frac{1}{n_T}\left(\frac{1}{\rho_T} + \frac{1}{1-\rho_T}\right) + \frac{1}{n_C}\left(\frac{1}{\rho_C} + \frac{1}{1-\rho_C}\right)\right)$$

We further let $\mu_\Delta = \log\left(\frac{\rho_T(1-\rho_C)}{\rho_C(1-\rho_T)}\right)$ and simplify the variance of $\log(\hat{OR})$,

$$\frac{1}{n_T}\left(\frac{1}{\rho_T}+\frac{1}{1-\rho_T}\right)+\frac{1}{n_C}\left(\frac{1}{\rho_C}+\frac{1}{1-\rho_C}\right)=\frac{1}{n}\left(\frac{1}{\rho_T(1-\rho_T)}+\frac{1}{\rho_C(1-\rho_C)}\right)$$

where we have assumed that $n_T = n_C = n$. Following the methodology proposed in the main paper, we aggregate historical data from sources $q = 1, ..., Q$ which has been summarized in the form $\lambda_q \dot{\sim} \mathcal{N}(\theta_q, \tau_q^2)$ (where λ_q now represent log-odds ratios) into a single prior for μ_Δ,

$$\mu_\Delta | \boldsymbol{y_1}, ..., \boldsymbol{y_Q} \dot{\sim} \mathcal{N}(\theta_{CP^*}, \sigma^2_{CP^*}),$$

where, as in the main paper,

$$\theta_{CP^*} = \sum_{q=1}^{Q} p_q^* \theta_q, \;\; \sigma^2_{CP^*} = \left(\sum_{q=1}^{Q} \xi_q^{-2}\right)^{-1},$$

$$p_q^* = \frac{\xi_q^{-2}}{\left(\sum_{q=1}^{Q}\xi_q^{-2}\right)},$$

$$\xi_q^{-2} = \left[\tau_q^2 + \frac{w_q b_{01}}{a_{01}-1} + \frac{(1-w_q)b_{02}}{a_{02}-1}\right]^{-1}.$$

The prior is updated to a posterior with the new trial data,

$$\mu_\Delta | \boldsymbol{y_1}, ..., \boldsymbol{y_Q}, \boldsymbol{y_{new}} \dot{\sim} \mathcal{N}\left(d_{\theta^*}, \sigma^2_{\theta^*}\right),$$

where

$$d_{\theta^*} = \frac{\sigma^{-2}_{CP^*}\cdot\theta_{CP^*} + \log(\hat{OR})\cdot n\left(\frac{1}{\rho_T(1-\rho_T)}+\frac{1}{\rho_C(1-\rho_C)}\right)^{-1}}{\sigma^{-2}_{CP^*} + n\left(\frac{1}{\rho_T(1-\rho_T)}+\frac{1}{\rho_C(1-\rho_C)}\right)^{-1}}$$

and

$$\sigma^2_{\theta^*} = \left(\sigma^{-2}_{CP^*} + n\left(\frac{1}{\rho_T(1-\rho_T)}+\frac{1}{\rho_C(1-\rho_C)}\right)^{-1}\right)^{-1}.$$

Applying the same decision criterion with a clinically meaningful effect size δ, the minimum sample size needed for (each arm in) the new study can be found as:

$$n \geq \left(\frac{1}{\rho_T(1-\rho_T)}+\frac{1}{\rho_C(1-\rho_C)}\right)\left(\left(\frac{z_\eta + z_\zeta}{\delta}\right)^2 - \sigma^{-2}_{CP^*}\right).$$

A.2 Bayesian sample size formula for a two-armed RCT with time-to-event endpoints

We now consider extending the proposed sample size formula for planning an RCT with a time-to-event outcome incorporating borrowing from historical data. As in the previous example, the derivation follows similar steps as the Supplementary Material in Zheng et al. (2023) with the same key differences already noted. For simplicity, we follow George and Desu (1974) to assume that event times from the new trial T_{ij} are exponentially distributed:

$$T_{ij} \sim \exp(\pi_j) \;\; i = 1, ..., n_q; \;\; j = E, C$$

with rate $\pi_j > 0$ (and the treatment arm now coded 'E' for clarity). The average time-to-event observed in treatment group j is $\bar{T}_j$ and the corresponding number of events is denoted D_j. By the central limit theorem,

$$\bar{T}_j \dot{\sim} \mathcal{N}\left(\frac{1}{\pi_j}, \frac{1}{D_j \pi_j}\right).$$

By the delta method,

$$\log(\bar{T}_j) \dot{\sim} \mathcal{N}\left(-\log(\pi_j), \frac{1}{D_j}\right),$$

therefore,

$$\log\left(\frac{\bar{T}_E}{\bar{T}_C}\right) \dot{\sim} \mathcal{N}\left(\log\left(\frac{\pi_E}{\pi_C}\right), \frac{1}{D_E} + \frac{1}{D_C}\right).$$

We further let $\mu_\Delta = \log\left(\frac{\pi_E}{\pi_C}\right)$. Following the methodology proposed in the main paper, we aggregate the historical data into a single prior for $\mu_\Delta | \boldsymbol{y_1}, ..., \boldsymbol{y_Q} \dot{\sim} \mathcal{N}(\theta_{CP^*}, \sigma^2_{CP^*})$. It is assumed that historical data from sources $q = 1, ..., Q$ has been summarized in the form $\lambda_q \dot{\sim} \mathcal{N}(\theta_q, \tau_q^2)$ (where λ_q now represent log ratios of mean event times),

$$\theta_{CP^*} = \sum_{q=1}^{Q} p_q^* \theta_q, \;\; \sigma^2_{CP^*} = \left(\sum_{q=1}^{Q} \xi_q^{-2}\right)^{-1},$$

$$p_q^* = \frac{\xi_q^{-2}}{\left(\sum_{q=1}^{Q} \xi_q^{-2}\right)},$$

$$\xi_q^{-2} = \left[\tau_q^2 + \frac{w_q b_{01}}{a_{01} - 1} + \frac{(1 - w_q) b_{02}}{a_{02} - 1}\right]^{-1}.$$

The prior is updated to a posterior with the new trial data,

$$\mu_\Delta | \boldsymbol{y_1}, ..., \boldsymbol{y_Q}, \boldsymbol{y_{new}} \dot{\sim} \mathcal{N}\left(d_{\theta^*}, \sigma^2_{\theta^*}\right),$$

where

$$d_{\theta^*} = \frac{\sigma^{-2}_{CP^*} \cdot \theta_{CP^*} + \log\left(\frac{\bar{T}_E}{\bar{T}_C}\right) \cdot (\frac{1}{D_E} + \frac{1}{D_C})^{-1}}{\sigma^{-2}_{CP^*} + (\frac{1}{D_E} + \frac{1}{D_C})^{-1}}$$

and

$$\sigma^2_{\theta^*} = \left(\sigma^{-2}_{CP^*} + \left(\frac{1}{D_E} + \frac{1}{D_C}\right)^{-1}\right)^{-1}.$$

Applying the same decision criterion as the main paper, the minimum sample size required in the new trial can be found according to

$$\frac{D_E D_C}{D_E + D_C} \geq \left(\frac{z_\eta + z_\zeta}{\delta}\right)^2 - \sigma^{-2}_{CP^*}.$$

Equivalently,

$$D \geq \frac{1}{R(1-R)}\left[\left(\frac{z_\eta + z_\zeta}{\delta}\right)^2 - \sigma^{-2}_{CP^*}\right],$$

where δ is a target clinically meaningful effect size, $D = D_E + D_C$ denotes the total number of events required and R is the proportion of the sample randomized to the treatment arm E. We note that further work would be needed to derive a sample size formula for time-to-event data with censoring.

A.3 Bayesian sample size formula for a single-arm trial with a binary outcome

In early phase oncology trials single-arm designs with binary outcomes are frequently conducted. Here we consider planning such a trial incorporating borrowing from historical data. Suppose in the new trial all patients are given a new treatment and observed to be either a 'responder' or 'non-responder' according to predetermined criteria. Letting Y denote the number of responders and n the number of patients needed in the new trial. This leads to $\mathbb{E}(Y) = np$ and $Var(Y) = np(1-p)$ where p is the proportion of responders and $p \, \epsilon \, [0, 1]$. By the delta method, the estimator of log-odds is asymptotically normally distributed,

$$\log\left(\frac{\hat{p}}{1-\hat{p}}\right) \dot{\sim} \mathcal{N}\left(\log\left(\frac{p}{1-p}\right),\, np(1-p)\right)$$

We further let $\mu_\Delta = \log\left(\frac{p}{1-p}\right)$. Following the methodology proposed in the main paper, we aggregate the historical data relating to the same arm into a single prior for $\mu_\Delta | \boldsymbol{y_1}, ..., \boldsymbol{y_Q} \dot{\sim} \mathcal{N}(\theta_{CP^*}, \sigma^2_{CP^*})$, where

$$\theta_{CP^*} = \sum_{q=1}^{Q} p_q^* \log\left(\frac{\hat{p}_q}{1-\hat{p}_q}\right),\ \sigma^2_{CP^*} = \left(\sum_{q=1}^{Q} \xi_q^{-2}\right)^{-1},$$

$$p_q^* = \frac{\xi_q^{-2}}{\left(\sum_{q=1}^{Q} \xi_q^{-2}\right)},$$

$$\xi_q^{-2} = \left[\tau_q^2 + \frac{w_q b_{01}}{a_{01}-1} + \frac{(1-w_q) b_{02}}{a_{02}-1}\right]^{-1}.$$

The prior is updated to a posterior with the new trial data,

$$\mu_\Delta | \boldsymbol{y_1}, ..., \boldsymbol{y_Q}, \boldsymbol{y_{new}} \dot{\sim} \mathcal{N}\left(d_{\theta^*}, \sigma^2_{\theta^*}\right),$$

where

$$d_{\theta^*} = \frac{\sigma^{-2}_{CP^*} \cdot \theta_{CP^*} + \log\left(\frac{\hat{p}}{1-\hat{p}}\right) \cdot (np(1-p))^{-1}}{\sigma^{-2}_{CP^*} + (np(1-p))^{-1}}$$

and

$$\sigma^2_{\theta^*} = \left(\sigma^{-2}_{CP^*} + (np(1-p))^{-1}\right)^{-1}.$$

Applying the same decision criterion with a clinically meaningful effect size δ, the minimum sample size needed for the new study can be found as:

$$n \geq \frac{1}{p(1-p)}\left[\left(\frac{z_\eta + z_\zeta}{\delta}\right)^2 - \sigma^{-2}_{CP^*}\right].$$

References

Agresti, A. (2003). *Categorical data analysis*. Wiley Series in Probability and Statistics. Wiley.

George, S. L. and Desu, M. (1974). Planning the size and duration of a clinical trial studying the time to some critical event. *Journal of chronic diseases*, 27(1-2):15–24.

Zheng, H., Grayling, M. J., Mozgunov, P., Jaki, T., and Wason, J. M. S. (2023). Bayesian sample size determination in basket trials borrowing information between subsets. *Biostatistics*, 24(4):1000 – 1016.
